# A Manual of Instruction for Enlisting and Discharging Soldiers: With Special Reference to the Medical Examination of Recruits, and the Detection of Disqalifying and Feigned Diseases

**Published:** 1863-11

**Authors:** 


A Manual of Instructions for Enlisting and Discharging Soldiers: with special reference to the Medical Examination of Recruits, and the Detection of Disqualifying and Feigned Diseases. By Roberts Bartholow, A.M., M.D., Assistant-Surgeon U. S. Army; Surgeon-in-Charge of McDougal General Hospital; Professor of Military Medical Jurisprudence, Army Medical School. Adopted by the Surgeon-General for issue to Medical Officers of the Army. Philadelphia: J. B. Lippincott & Co. 1863.

This is a small-sized octavo volume of 276 pages, published in the well-known neat and attractive style of Lippincott & Co. The scope and objects of the author are pretty well indicated in the titlepage, as copied above. The work is divided into the four following sections:—

"Real Disqualifications for Military Service."

"Pretended Disqualifications for Military Service."

"Enlisting Soldiers."

"Discharging Soldiers."

From a very hasty glance at the matter and style of this work, we should think it sufficiently full and plain for all practical purposes. To those who are engaged in examining men, either as candidates for enlistment into, or discharge from, the Army, it is an exceedingly convenient manual; while it will be found useful for reference in the library of every practitioner. For sale by S. C. Griggs & Co., Lake Street, Chicago.

